# Addressing an Identified Need: Training in Serious Incidents Investigations and Coroner's Inquests for Psychiatric Trainees in Kent and Medway NHS and Social Care Partnership Trust (KMPT)

**DOI:** 10.1192/bjo.2024.317

**Published:** 2024-08-01

**Authors:** Maria Moisan, Verity Williams, Segun Ayanda, Rachel Daly, Koravangattu Valsraj

**Affiliations:** Kent and Medway NHS and Social Care Partnership Trust, Dartford, United Kingdom

## Abstract

**Aims:**

Navigating a Serious Incident (SI) investigation and participating in a Coroner's Court proceedings can pose challenges for psychiatry trainees. The Higher Training curriculum emphasizes active participation in activities that enhance patient safety and care quality. This project aims to enhance patient safety and trainee confidence by improving training on SI investigations and Coroners Court proceedings.

**Methods:**

Using Quality Improvement (QI) methodology, in the first cycle an initial survey was distributed to all psychiatry trainees and middle grade doctors working in Kent and Medway (n = 67) to establish baseline knowledge and confidence levels in areas related to risk assessment & management, SI investigations and Coroner's Inquests.

In response to the identified need for training, we organized the Initial Training Event with support from Deputy Chief Medical Officer for Quality and Safety, Patient Safety Team and Medical Education Department. The half-day, in-person event was opened to all doctors and featured 5 sessions: Serious Incident Investigation Process, Thematic Review of Suicides, Systems Engineering and Human Factors in Patient Safety, Learning from Mortality and Structured Judgement Review along with ‘Being Involved in Investigation – An Investigator's Guide’. Data from a survey of attendees (n = 47) informed the development of a tailored training session for psychiatry Core and Higher Trainees.

**Results:**

The initial survey received 32 responses (response rate: 47.76%). 71.88% of respondents had little to no understanding of SI investigation processes. Remarkably, 87.5% expressed strong interest in receiving training on conducting SI investigations. 90.62% were extremely or very interested in receiving training on participating in a Coroner's Inquest.

47 doctors attended the Initial Training Event. 30 responded to the feedback questionnaire (47.76%). All doctors found the training useful, with over 90% rating it ‘very’ or ‘extremely’ useful. 97% felt that the training would improve their clinical practice in terms of patients’ safety. After the training, 60% understood the process of conducting an SI investigation a moderate amount; 33.33% understood the process a lot or to a great extent. Nevertheless 92.86% felt a need for additional training in SI investigations. 63.33% suggested making training available yearly, and 36.67% favoured making it mandatory training.

**Conclusion:**

This project identified a significant need for training in SI investigations and Coroner's Court proceedings among psychiatric trainees. An Initial Training Event developed from the first QI cycle survey data received positive feedback. The next phase involves developing a tailored training program that addresses identified knowledge gaps. Further considerations include making this training a regular event.

